# Artificial intelligence in HIV research: a structured review and task-oriented clinical framework

**DOI:** 10.3389/fdgth.2026.1863792

**Published:** 2026-07-23

**Authors:** Ruben E. Munoz-Cabrera, Joaquin Bravo-Urbieta, Raquel Martinez-España, Sergio Aleman Belando, Jose Miguel Gomez-Verdu, Enrique Bernal-Morell, Jose M. Juarez

**Affiliations:** 1 Med AI Lab, University of Murcia, Murcia, Spain; 2 Murcian Bio-Health Institute (IMIB-Arrixaca), Murcia, Spain; 3 Section of Infectious Diseases, Morales Meseguer University Hospital, Murcia, Spain; 4 Section of Infectious Diseases, Reina Sofía University Hospital, Murcia, Spain

**Keywords:** artificial intelligence, clinical decision support, deep learning, digital health, healthcare informatics, HIV, machine learning

## Abstract

**Background:**

Human Immunodeficiency Virus (HIV) poses a global health challenge despite the success of the Antiretroviral Treatment (ART), which allows the disease to be potentially controlled. The growing availability of heterogeneous data has impulsed the use of Artificial Intelligence (AI) to address the different clinical domains of HIV, facilitating the creation of decision making tools to assist clinical professionals.

**Objective:**

This study aims to propose a task-oriented framework to support clinicians and researchers that want to apply AI in the management of HIV, linking clinical tasks with appropriate AI approaches.

**Methods:**

Following a structured review of over 50 relevant studies in this area since 2017 to November 2025, literature was analysed considering four complementary dimensions: (1) the scope of clinical application in the context of HIV, (2) the data types employed, (3) databases and data sources, and (4) AI techniques used, ranging from statistical models to approaches based on neural networks and natural language.

**Results:**

Building on this analysis, the proposed framework associates the main clinical tasks of the different clinical domains of HIV with the most appropriate AI approaches, providing recommendations of algorithms and techniques that can be used in different scenarios. Ultimately, this study tackles the use of AI as a key tool for HIV management in different phases of the disease, taking into account the type of available data.

**Conclusions:**

AI represents a key tool for enhancing HIV management. This framework provides a structured basis for future research, though it will be necessary to continuously redefine and update this framework as new medical challenges and technical methods arise.

## 1 Introduction

According to the World Health Organization (WHO) [1], the Human Immunodeficiency Virus (HIV) remains a global public health problem. This retrovirus primarily targets CD4-positive T lymphocytes, which its progressive depletion drives the development of immunodeficiency in people living with HIV (PLWH). If not treated properly, this infection can lead to Acquired Immunodeficiency Syndrome (AIDS) and death, so early diagnosis and prompt initiation of effective Antiretroviral Treatment (ART) are essential. Currently, ART has made it possible to turn HIV into a manageable chronic infection, stopping or reverting the progression of the disease to AIDS, as well as the appearance of other pathologies. Beyond its individual clinical benefits, effective antiretroviral therapy confers substantial public health advantages. Sustained viral suppression eliminates the risk of sexual HIV transmission and prevents mother-to-child transmission. Together, these effects form the basis of the Treatment as Prevention (TasP) strategy and reinforce broader prevention approaches, including pre-exposure prophylaxis (PrEP). However, new clinical challenges have emerged, such as Low Level Viremia (LLV) [2, 3] or some non-AIDS defining comorbidities, which require additional tools to facilitate the management of more complex cases.

In recent years, there has been a constant growth in the volume of data, both structured (e.g., longitudinal clinical variables, biomarkers, or genomic information) and unstructured (e.g., clinical notes). The complexity of heterogeneous data, characterized by non-linear relationships or high dimensionality [4], makes that, in many scenarios, classical statistical techniques are unable to adequately capture the necessary information for analysis and management of the clinical progression of PLWH.

In this sense, Artificial Intelligence (AI) techniques emerge as key tools to deal with complex data [5]. Both machine learning and deep learning techniques are able to model complex relationships and identify patterns in the data, thus facilitating the different clinical domains of the disease: prevention, diagnosis, treatment, progression and comorbidities. In order to properly address the diversity of techniques, data and clinical applications, it is necessary to contextualize the use of these techniques in the HIV field in a structured way.

The purpose of this study is to provide a broad overview of the impact of AI in the context of HIV, by analysing both the clinical application of techniques and the data types and approaches used. The aim is not only to synthesise existing literature, but also to develop a practical approach in form of a task-oriented framework which clinicians and researchers can rely on to select the most appropriate technique according to the problem at hand.

Our study is organized progressively culminating in a task-oriented framework. Firstly, the basic principles of AI techniques used in the field of HIV are introduced. The process of identifying and selecting relevant studies for analysis and overview is then defined. This analysis considers the scope, types of data and databases and AI approaches used. Finally, the proposed framework integrates these elements to provide recommendations in order to help clinicians and researchers make decisions in future studies.

## 2 Basic principles of AI

This section briefly introduces the concepts needed to contextualize the AI techniques presented in the following sections.

**Machine Learning (ML)** involves discovering patterns in data through algorithms, using these to perform some specific task [6]. Depending on the predictive model used, we can classify [7] the ML techniques as follows:

- Supervised ML: Uses a set of labelled data to learn a function that relates inputs and outputs.
- Unsupervised ML: It is used when we do not have labelled data, highlighting the clustering problem, consists of organizing a collection of elements into a set of homogeneous groups.
- Semi-supervised ML: Combines both approaches using a small amount of labelled data next to a large volume of unlabelled data.
- Reinforcement learning: It is based on an agent’s interaction in an environment. This agent must learn the optimal policy to maximize the accrued reward.

Besides this, we find **Deep Learning (DL)**, which is a subdiscipline of ML that uses deep neural networks, able to learn hierarchical and abstract representations of data [8]. Among the main DL based architectures we find Deep Neural Networks (DNN), Multilayer Perceptron (MLP) or Recurrent Neural Networks (RNN), which includes Long Short-Term Memory Networks (LSTMs).

When we want to extract information from unstructured data as text, we find the techniques of **Natural Language Processing (NLP)**, a set of computational techniques capable of analysing and interpreting human language [9].

In recent years, due to the advance of DL, **Large Language Models (LLMs)** have emerged, which constitute a particular class of deep learning models based on transformers. These models, trained on large text corpus, are able to understand, generate and reason natural language [10].

## 3 Methods

### 3.1 Study design

This study is conducted as a structured literature review aimed at synthesising current evidence on the application of artificial intelligence across the HIV care continuum and developing a task-oriented framework to support the selection of AI methodologies for different clinical problems. Rather than performing a formal systematic review, the objective is to integrate representative evidence from the literature and organise it according to clinically relevant dimensions.

To enhance transparency and reproducibility, the literature search process has been strengthened by incorporating relevant recommendations from the PRISMA-S reporting guideline.

### 3.2 Literature research

#### 3.2.1 Information sources

We began by establishing the data sources used, and in order to analyse the existing literature the following databases and repositories have been consulted: ScienceDirect, PubMed, Cochrane Database, IEEE, AIDS journal and Google Scholar. Each database was searched independently using its native search interface. No multi-database search platforms were used.

No study registries were searched, and no additional website browsing or author contacts were performed as part of the search process. No other supplementary search methods were employed.

The studies considered were published between 2017 and 2025, most of them being from 2022 onwards. The search was carried out between October and November 2025 in several iterations.

#### 3.2.2 Search strategy

The search strategy combined terms related to artificial intelligence with terms associated with HIV infection adapted to the syntax of each database.

The search terms used were combinations of specific terms related to AI and its techniques such as “artificial intelligence,” “machine learning,” “deep learning,” “chatbot,” “natural language processing” or its abbreviations “**ai**,” “**ml**,” “**dl**,” “**nlp**”; and HIV or AIDS terms such as “human immunodeficiency virus” and abbreviations such as “**hiv**” or “**aids**”.

Details of the search strings are shown in Table 1.

**Table 1:** Search strings employed for each database during the literature search process.

Database	Search string
Science Direct	[terms] “hiv,” “human immunodeficiency virus,” “aids,” “machine learning,” “artificial intelligence”
PubMed	(“artificial intelligence” OR “ai” OR “machine learning” OR “ml” OR “deep learning” OR “dl” OR “natural language processing” OR “nlp” OR “artificial intelligence review”) AND (“hiv”[Title/Abstract])
Cochrane	(“artificial intelligence” OR “ai” OR “machine learning” OR “ml” OR “deep learning” OR “dl” OR “chatbot” OR “natural language processing” OR “nlp”) AND (“hiv” OR “human immunodeficiency virus”)
IEEE Xplore	“artificial intelligence” AND “hiv”
AIDS	Manual search of relevant articles published in the journal AIDS using keyword screening (“HIV,” “artificial intelligence,” “machine learning”)
Google Scholar	(“artificial intelligence” OR “ai” OR “natural language processing” OR “nlp” OR “chatbot”) AND (“hiv”)

No database-level search filters regarding publication year or language were applied. English language was considered during study selection as eligibility criteria.

The search strategy has been developed specifically for this study and was not adopted from previous reviews.

To maximise coverage, we manually added some references of the most relevant studies, identifying new publications that were not found in the initial electronic database search.

### 3.3 Elegibility criteria

In order to ensure that the included literature was relevant to the application of AI in HIV research, eligibility criteria was first defined.

Inclusion Criteria were:

1. Research articles written in English.
2. Original studies applying AI methods on data directly related to HIV in one or more of its clinical domains: prevention, diagnosis, treatment and progression of the disease.
3. Poblational or methodological studies that used exclusively PLWH to analyse possible comorbidities and the consequences of the disease.
4. Reviews on the application of AI in HIV disease in order to see the extent of these and their limitations.

As Exclusion Criteria were selected:

1. Studies not written in English.
2. Original studies or reviews where the central theme or data used are not related to HIV specifically.

### 3.4 Study selection

The study selection process was supported by a reference management workflow tool using Zotero, which was used to organise all retrieved records and assist in the identification of duplicate entries.

Titles and abstracts were screened to assess relevance to the application of artificial intelligence in HIV research. Subsequently, full-text articles were evaluated against the eligibility criteria defined in Section 3.3. Those that met the inclusion criteria were included, while those meeting at least one exclusion criterion were excluded.

The selection process was iterative in nature, incorporating both database search results and additional relevant studies identified through reference list screening of key articles.

### 3.5 Study synthesis

Having finalized the search process, 52 studies were found that met the inclusion criteria. Table 2 shows the distribution of these studies grouped into literature reviews and then the different AI approaches.

**Table 2:** Number of studies included in our overview.

Approach	Quantity
Literature reviews	3
Statistical Approaches	4
ML	21
DL	9
NLP/Chatbot	11
LLM	4

This overview follows a narrative approach due to the heterogeneity of the studies, their different data types and application domains in the HIV context.

In order to organize the different studies, we evaluate: (i) the clinical application to which they are directed, (ii) data types, (iii) databases, and (iv) the AI techniques used to process the data.

## 4 Results

**Figure 1:**
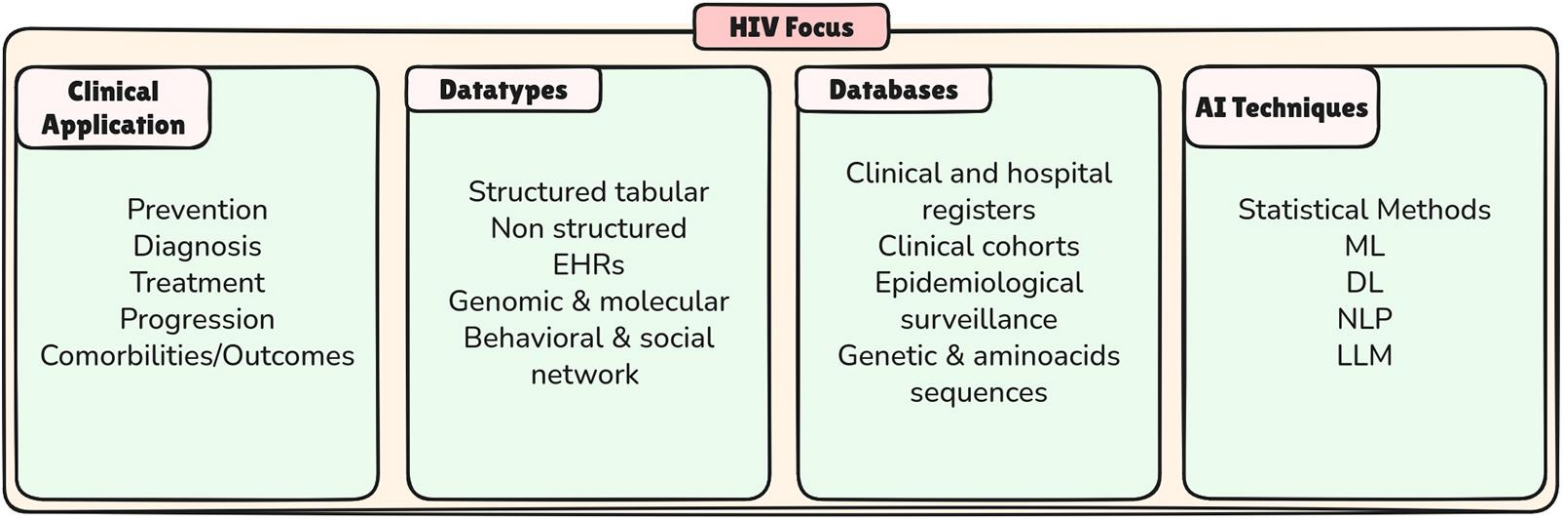
Dimensions studied in the overview.

In Figure 1 we can see how this review is structured, taking into account four main dimensions:

1. The scope of clinical application in the HIV context where different AI techniques are used.
2. The data types used in the various studies.
3. The datasources used in evaluating the databases.
4. The AI techniques employed, considering the clinical application and datatypes in order to identify patterns in data, as well the most appropriate approach in each situation.

Finally, based on this analysis, a task-oriented framework is developed to guide the selection of appropriate techniques according to the medical field and the type of data.

### 4.1 Clinical application

The studies analysed address the phases of clinical management of HIV: prevention, diagnosis, treatment, disease progression, and possible comorbidities or associated pathologies. This is intended to address the question of where AI is applied in different areas of HIV, providing a structured framework for further analysis.

#### 4.1.1 Prevention

This phase of HIV clinical management focuses on identifying people with a high risk of contracting the disease, as well as promoting prophylactic strategies such as Preexposure Prophylaxis (PrEP), and analysing social behaviours to reduce virus transmission.

In this area we find most of the studies analyzed. The different applications of AI can be seen:

- Risk identification : Krakower et al. [11] develops and validates a ML based model to identify PrEP candidates using more than 20 predictor variables in structured data from Electronic Health Records (EHRs). Kakalou et al. [12] identify and examine perceptions and trends related to PrEP discussed on Twitter using techniques such as text mining.
- Identification of socio-behavioural factors: Through an unsupervised learning paradigm, Mutai et al. [13] identify the homogeneity of South African countries in HIV based on sociobehavioral patterns by finding two clusters by sex where people from each cluster shared similar relevant characteristics.
- Use of chatbots and natural language processing: Chatbots such as Amanda Selfie [14] or SHIHbot [15] have been developed to create demand for PrEP and provide sexual health information. NLP techniques have also been applied to develop a chatbot in Amharic, a language used in Ethiopia [16] designed for HIV awareness and counselling.
- Networking Contacts: Through the use of graph-based convolutional neural networks and a semi-supervised approach, Xiang et al. [17] is able to detect previously unknown HIV infections within a network of contacts. Ni et al. [18] evaluate the effectiveness of a ML model for identifying sexual health influencers among men who have sex with men (MSM) in China, in order to promote secondary distribution of HIV self-test kits (SD-HIVST).

#### 4.1.2 Diagnosis

This area focuses on using AI to improve the accuracy and efficiency of HIV detection. The studies analysed address different perspectives within this domain:

- Detection of suspicious cases: Through the use of text mining and ML techniques, Morales-Sánchez et al. [19] succeed in improving the diagnosis of HIV through a LLM for the biomedical clinical domain in Spanish, which analyses clinical notes to classify patients into suspects and non-suspects.
- Autotest interpretation : Ngcobo et al. [20] find several studies where artificial vision techniques are used to interpret fast HIV tests achieving results very close to 100% and surpassing human interpretation.
- HIV screening: Alehegn [21] employs ML and DL techniques to improve HIV prediction achieving very significant results with high precision.
- Chatbots for self-diagnosis: Clinical trials like the one by Chen et al. [22, 23] evaluate the effectiveness of a NLP-designed chatbot to provide real-time support during HIV self-testing.

#### 4.1.3 Treatment

This area deals with the prediction of adherence to ART, the identification of virological failure or treatment management through tools such as chatbots:

- Virological failure (VF) prediction : Saboka et al. [24] employs several supervised ML algorithms to predict viral load and adherence level in PLWH under ART. In these models, variables such as treatment change of regimen or its duration are identified as relevant predictors of virological failure.
- Adherence monitoring to ART : Yenekal et al. [25] uses ML in data from patients on antiretroviral treatment at a hospital in Ethiopia to assess adherence. Age, area of residence and WHO status are significantly associated with adherence to treatment, showing that as age increases, adherence to ART decreases. Another study by Najjuuko et al. [26] applies ML to predict the risk of low adherence to ART among adolescents with HIV, identifying different risk factors and protectors against poor adherence to ART.
- Drug resistance: Riveros et al. [27] characterised resistance to protease inhibitors in HIV-1 by evaluating multiple ML and DL models.
- Chatbots as support tools : There are chatbots like MARVIN [28], designed to promote HIV self-control and self-management.

#### 4.1.4 Progression

This area evaluates the progression of HIV to AIDS and analysis of longitudinal patterns associated with disease evolution such as persistent low-grade viraemia in people living with HIV:

- Prediction of HIV to AIDS progression: Mao et al. [29] develops and compares ML models that allow create a nomogram to identify risk factors associated with the progression of HIV to AIDS, being the most important predictors CD4 levels and the route of disease transmission.
- Development of LLV in PLWH : There are several studies [30, 31] that analyse the effect of LLV in patients under ART in order to assess the risk of virological failure.
- Progression modelling: Through clustering techniques, Lynch et al. [32] group patients that share common patterns of their biomarkers. Using ensemble clustering 4 groups of patients were found, highlighting the relationship between low CD4 levels and high levels of viral load (CV).

#### 4.1.5 Comorbidities and outcomes

This clinical domain covers possible chronic comorbidities, co-infections and other pathologies frequently related to HIV:

- Chronic comorbidities: Russom et al. [33] apply ML and DL techniques to predict the occurrence of twelve comorbidities, evaluating the effect of including demographic variables along with their own laboratory values in the model. Ding et al. [34] predicts the risk of hyperlipidemia in people with HIV who have received High Activity Antiretroviral Therapy for 6 months, to help early intervention that prevents this disease.
- Mental health diseases and substance use. Mental health disorders and substance use have been analysed through natural language techniques applied in clinical notes. Ridgway et al. [35] applies this approach to identify these pathologies in PLWH, thus facilitating the clinical management of the disease.
- Co-infections or associated pathologies. In Uganda, a study develops an ML [36] model to detect possible cervical cancer related lesions in women under antiretroviral treatment. Another study by Murnane et al. [37] evaluates whether ML algorithms using routinely collected data could predict viremia in women with HIV during the postpartum period in Sub-Saharan Africa.

### 4.2 Data types and databases

#### 4.2.1 Data types

The studies analysed include data of different nature, conditioning both the clinical objectives and the artificial intelligence techniques that can be used.

- **Structured clinical data (tabular)**: These are the most common data types in the literature analysed, including demographic variables (age, sex, etc.), laboratory results (such as CD4 levels and viral load), treatment history, and data related to clinical events.
- **Unstructured clinical data**: Clinicians collect textual information through clinical notes. This type of unstructured information may be able to capture determinant factors of health as well as circumstances and behaviours in people with HIV as reported by the study of Chen et al. [38].
- **Electronic Health Records (EHRs)** : These records contain both structured information (laboratory clinical values) and unstructured information (clinical notes). In addition, they allow the capture of complex information, although their heterogeneity presents a challenge when it comes to applying artificial intelligence techniques. Studies such as Feller et al. [39] looked at both structured and unstructured fields, showing that together they can help improve prediction in HIV diagnosis.
- **Genomic and molecular data**: This includes high-dimensional molecular-level data that characterizes biological processes at different layers of organization. Some studies include sequences of amino acids or nucleotides that serve as a basis for tasks such as the classification of HIV-1 group M subtypes [40], where applying DL, it is possible to classify the 12 subtypes of group M, facilitating the design of vaccines and pharmaceuticals. Another study, by Danaila et al. [41], aims to predict whether an HIV virus is susceptible to a monoclonal antibody using amino acid sequences and using a deep learning approach.
- **Social media and socio-behavioural data**: This data comes from population surveys or social networks. Studies such as Kakalou et al. [12] use tweets to identify and examine perceptions and trends related to PrEP using text mining techniques. Twitter (now X) can also identify HIV-related influencers by applying DL [42]. A different approach is the one of Yu et al. [43], which investigates different deep models to predict the state of HIV in high-risk population using network information.

#### 4.2.2 Datasources and databases

Here we analyse the sources of data used in most studies, finding a variety of databases:

- **Hospital and clinic records**: Most studies use data from a single institution, specifically in US or Chinese hospitals, such as the studies by Goyal et al. [44] and Zhan et al. [45], where they use data from a hospital in San Diego and Nanning, respectively, to carry out their studies. Also, we found multicentre studies such as one conducted using EHRs data from 17 hospitals in Indonesia to improve HIV prediction using DL [46].
- **Specialized clinical cohorts**: We find several studies using the Swiss HIV Cohort Study (SHCS) [31, 47] applying both unsupervised learning methods and classical statistical approaches. Another cohort employed is the Spanish AIDS Research Network Cohort (CoRIS), used in a study to find rules of association using a semi-supervised approach [48].
- **Epidemiological surveillance data**: Several studies by Mutai et al. [13, 49] use data from the Population-based HIV Impact Assessment project (PHIA), which surveys up to 14 countries with key HIV factors, facilitating the screening of patients and the identification of socio-behavioural factors in these people.
- **Genomic data**: Genetic and amino acid sequences are obtained from databases such as *HIV Sequence Database* from Los Álamos [40] or *Stanford University HIV Drug Resistance Database* [27]. The CATNAP database is also used for antibody tests [41].

#### 4.2.3 Data type determines the technique

Diversity and heterogeneity of data in different studies highlight the importance of choosing an AI technique that suits the data type we want to work with. This is why we want to analyse how the methodological selection of algorithms is conditioned by the data.

- ML models are used repeatedly in structured (tabular) data for their efficiency and interpretability as pointed out by several reviews [50, 51], where their potential is indicated over classical statistical approaches and other approaches to predicting the risk of acquiring HIV [52].
- For processing of clinical notes and social media analysis, it is necessary to employ NLP techniques focused on prevention [12, 39] and also treatment [53].
- The rise in the use of LLMs has enabled the processing of large volumes of unstructured data (like text). In this context, two approaches emerge. The first is to adapt models already designed with ones own clinical notes as in the study by Zhou et al. [54], where, from a Google LLM, achieves a model in Dutch that facilitates the screening of patients for further testing. Another completely different approach is to evaluate the responses of generalist models such as ChatGPT or Gemini to HIV-related questions analysing their accuracy, reliability and reproducibility [55, 56].
- DL techniques that employ neural networks (DNN, RNN, etc.) are mainly applied in studies using amino acid sequences [41] or in studies with genomic sequences such as that of Hu et al. [57], where it develops a framework based on an attention mechanism, capable of predicting HIV integration zones. A systematic review also reports its use in other areas such as imaging to diagnose tuberculosis in people with HIV [20].

#### 4.2.4 Limitations and challenges in data

Despite the wide variety of data used, studies reveal a number of limitations in dealing with these data.

Firstly, most studies are conducted with limited sets of data or obtained in a single clinical centre, making it difficult to generalize the models obtained. Another recurring problem is class imbalance, particularly in studies related to adherence to ART, the occurrence of rare events [24, 25] or in the classification of gene sequences of subtypes of HIV, where some subtypes are clearly predominant [40]. This problem requires the use of techniques such as SMOTE, which can sometimes lead to bias in predictions, generating models with apparently high performance whose usefulness in real scenarios is limited.

An important aspect highlighted by a large number of studies and also pointed out by some reviews [20, 58], is the need for data interoperability, highlighting that it is appropriate to implement ontologies and have international HIV cohorts. This, together with the need to equip models with explainability techniques, would make it possible to facilitate understanding by clinical professionals as well as ensuring transparency of the models.

Finally, we find the problem of bias in data from a dual perspective. Firstly, the majority of studies are concentrated in the US, China and Africa, so that over-representation makes it difficult to generalise the use of models to other regions such as Europe. Lastly, we find a certain bias in the population groups targeted by the studies, which, as indicated by some reviews [58] are aimed at men who have sex with other men [17, 47], even though we have been able to find studies covering women or the general population [36, 49].

Overall, these limitations suggest that it would be useful to have a structured framework to help AI researchers in the selection of appropriate techniques for the clinical context of HIV.

### 4.3 AI and data analysis methods

**Figure 2:**
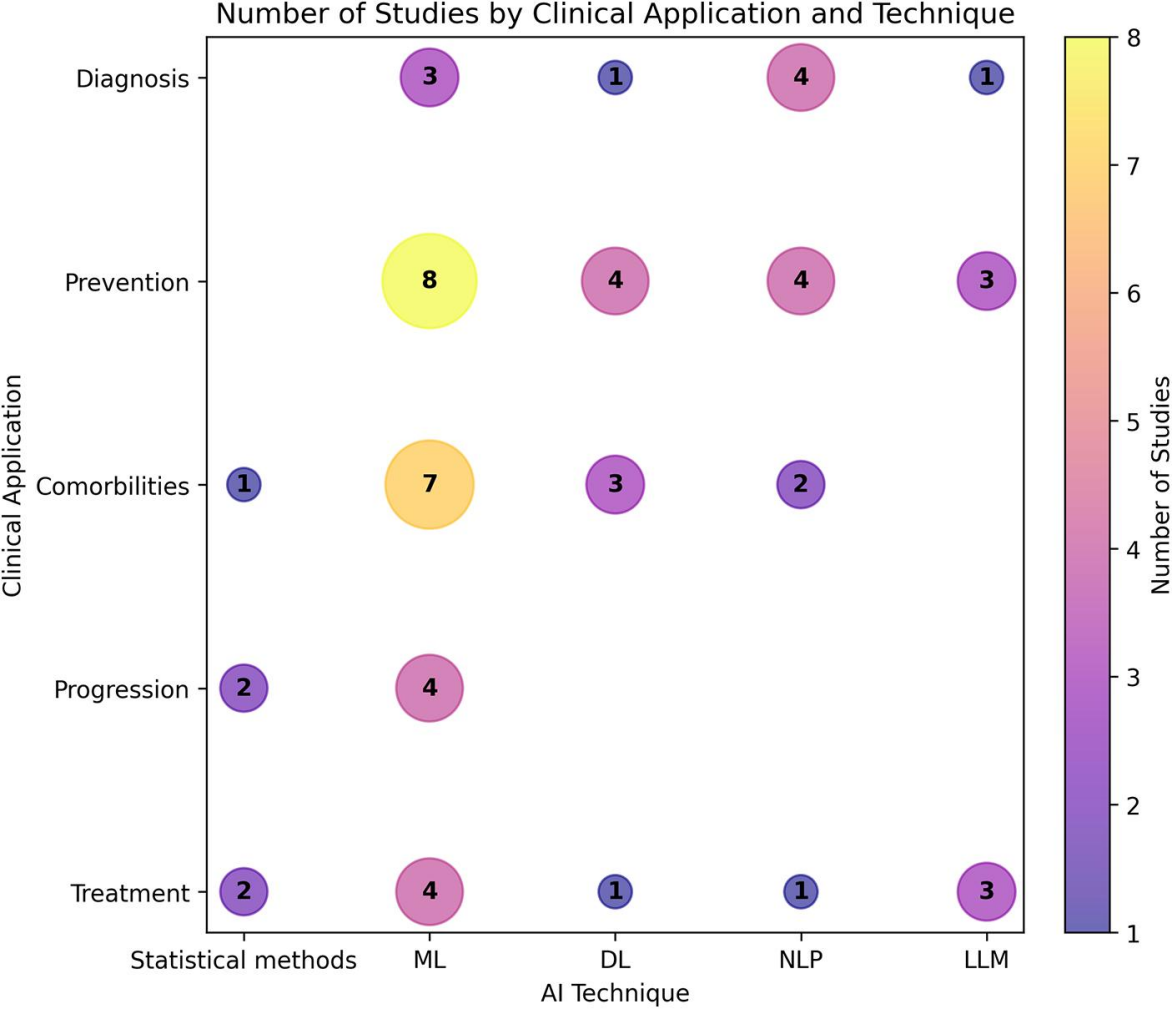
Bubble chart showing the number of studies relating each clinical domain of HIV and AI techniques.

In this last dimension we analyse the AI approaches applied in the different studies. In addition, we see that these approaches are widely applied to the different areas of clinical application, as we can see in the bubble chart in Figure 2, where bigger bubbles represent that there are more studies that apply a certain AI approach in a specific HIV clinical domain.

#### 4.3.1 Statistical approaches

Classical statistical approaches are based on the data distribution under parametric models. In the HIV context, proportional risk models are the most often used to model the risk of a particular event.

In the area of antiretroviral treatment (ART), Zhang et al. [59] explore risk factors associated with LLV after ART, defined as confirmed viral load values ranging from 50 to 999 copies/mL, by developing a predictive risk model visualized with a nomogram. This 11-year retrospective study uses data from people living with HIV at the Hangzhou Xixi Hospital in China. To do this, it uses a multivariate Cox regression along with Lasso to choose the optimal predictors. Six variables were identified as being significantly associated with LLV, including age, delayed ART and CD4 levels. A final nomogram assigns a score to each variable based on its standardized regression coefficient. This score indicates a patient’s probability of having LLV.

Alternatively, in the comorbidities domain, a longitudinal study by Tao et al. [60] assesses whether the presence of persistent LLV under ART increases the incidence of Diabetes Mellitus (DM) in people living with HIV in China. This study uses data from 8,731 patients under ART and without DM at baseline, with a follow-up of up to 7 years. To assess the risk of DM, a Cox regression model is also applied, adjusted for more than 15 sociodemographic and clinical variables. In addition, through a multivariate linear model it is possible to identify two different trajectories of blood glucose and fasting over time: one group with “stable” values and another group with a “rapid increase” in the levels. The latter group containing a significant percentage of patients with LLV.

Through these studies, we see that the application of statistical approaches such as Cox’s regression allows us to model the risk of a clinical event. This approach is suitable for longitudinal data in HIV, facilitating time modelling up to a certain event, as well as the creation of interpretable tools such as nomograms. However, given non-linear relationships in the data, a proportional risk model may be limited to when it comes to capturing complex patterns, requiring the use of machine learning techniques that allow more complex scenarios to be modelled.

#### 4.3.2 Machine learning

These methods are the most widely used approach in the studies analysed, particularly those which work with structured data. The most relevant studies are:

- Supervised ML.
  In the prevention domain, we find several studies that develop supervised models to identify patients at high risk of acquiring HIV, and therefore, candidates for pre-exposure prophylaxis (PrEP). Krakower et al. [11] and Marcus et al. [50] employ similar approaches using EHRs from large cohorts in Massachusetts and California, respectively. Both studies apply regularized regression with LASSO to select the most relevant predictors.
  Krakower et al. [11] evaluate more than 40 ML and regression configurations, including Lasso, Ridge, Random Forest (RF) or Support Vector Machine (SVM). The best model was LASSO, which reduced from 134 predictors to only 23 in the final model, obtaining Area Under the Curve (AUC) values higher than 0.85 in development and prospective validation and 0.77 in external validation. Marcus et al. [61], for their part, develop a very similar study on a study cohort of about 3.7 million patients. Applying logistic regression (LR) with LASSO, it reduces from 81 to 44 predictors in the final model, achieving an AUC of 0.84 in the validation set. In both studies, male sex, MSM status (men in relationships with other men) and residence in an area with a high incidence were highlighted as relevant predictors.
  Kagendi [62] presents a different study, aimed at predicting high viral load health facilities (hotspots), defined as centres where more than 20% of PLWH have an unsuppressed viral load. To develop the model, 4 million CV test records from the National AIDS & STI Control Program (NASCOP) repository are used, linked to geographic and administrative data from the “Kenya Master Health Facility List.” For modelling, CV was classified as suppressed CV (VLS), combining the categories LDL (CV ≤ 400 copies/mL) and LLV (CV 400–1,000 copies/mL) and, in unsuppressed CV, HDL (CV ≥ 1,000 copies/mL). The dataset was unbalanced with 70% of “non-hotspots.” The model developed was an RF with a stratified division of 75/25, and discriminated centres with an AUC of around 0.86 in the test and validation sets. Regions in northern Kenya were identified with a higher probability of having a hotspot, noting that they had less population and disease monitoring, and more civil conflicts.
  To close this section we see a study applied to the prediction of comorbidities. Russom et al. [33] evaluates four ML models (LR, XGBoost, RF and LGBM) and two DL models (MLP and TabNet) to produce a multi-tag prediction of twelve comorbidities (ICD-10 categories). It uses data from 2,200 patients at the South East London Hospital between 2012 and 2023 with 30 laboratory markers and 7 demographic and social attributes. For the modelling, it evaluates two configurations: one without demographic variables (unaware), and another that also considers laboratory markers (aware). Aware models consistently outperformed unaware models in predicting most comorbidities. The only exception was the category “Factors affecting health.” The final model is an XGBoost that achieves a 45.8 F1 macro in the aware model. In addition, these models improved the identification of minority categories.
- Unsupervised ML.
  In the HIV context, there is a clear willingness to use hierarchical clustering to find groups of patients with common characteristics.
  Several studies use clustering to determine disease progression based on the longitudinal profile of CD4 and VL. An example is the study by Farooq et al. [63], that designs a centroid-based algorithm to group patients according to the temporal dynamics of VL. Using data from 1,576 patients of the Rochester Medical Center’s and incorporating demographic and clinical predictors, it applies hierarchical clustering on the characteristics that define the dynamics of VL over time. It finds five groups of patients based on their viral load by distinguishing suppressed, low and high loads. To facilitate clinical interpretation of clusters, CMuRN (Centroid Methods using Radial Normalization) algorithm was designed, which, although not superior to complex ML models such as SVM or RF, is a highly interpretable technique.
  In another study, Mutai et al. [13] also uses hierarchical clustering to characterize the homogeneity of sub-Saharan African countries based on socio-behavioural predictors of more than 300,000 men and women from the Population-based HIV Impact Assessment (PHIA) Project. Starting from 20 attributes in men and 26 in women, it applies the Principal Component Analysis (PCA) reducing in each of the sexes to only 2 dimensions, explaining these 70.1% of the variance in men and 62.3% in women. Subsequently, applies hierarchical agglomerative clustering using the Euclidean distance and the Silhouette coefficient to choose the optimal number of clusters. Results show that in men, most descriptive characteristics were circumcision and residence, while in women, were residence and marriage. In both genders two clusters were found, one with people living and unemployed in rural areas and another with people living and working in urban areas. The high prevalence of HIV in women was also observed in both clusters, and a low prevalence in circumcised men. This highlights the relevance of socio-behavioural factors when analysing the risk of disease infection.
- Semi-supervised ML.
  This approach is the least prevalent in the context of HIV.
  The study by Araujo et al. [48] stands out, based on a minimum amount of data annotated by experts, is able to develop a system that filters rules of association (ARs) in the Spanish AIDS Cohort CoRIS. First, ARs of CoRIS diseases were extracted using the FP-Growth algorithm to classify them as relevant (True) or not relevant (False). Of these, 1,000 were manually reviewed by creating an expert-annotated training subset called ARAIDS. Finally, the semi-structured EXTRAE algorithm was applied and adapted, combining supervised (Random Forest) and unsupervised learning (Fisher’s exact test). This study revealed multiple HIV-related ARs such as the relationship between cardiovascular disease, diabetes and cancer with depression or psychosis. Also, other plausible relationships were found to be included in future research.

Overall, we observe that ML techniques are predominant in HIV prevention tasks and in the prediction of possible comorbidities. Compared to classical methods, they represent an advance in the ability to model non-linear relationships when dimensionality increases, resulting appropriate for structured tabular data. Among ML techniques, tree-based techniques such as Random Forest or ensemble methods like XGBoost show robust and efficient performance when we have labelled data, while hierarchical clustering is the option chosen when we do not have previous information. In addition, mechanisms of interpretability facilitate their clinical utility. However, when we face unstructured data or more complex structures such as gene sequences or text, there is a need to employ deep neural network approaches.

#### 4.3.3 Deep learning

Deep architectures allow the possibility of working with more complex data, such as unstructured data or longitudinal sequences. In the analysed studies, we observe the use of neural networks in genomic classification tasks or screening patients using longitudinal data.

Peng [40] designs a DL-based method for classifying the 12 subtypes of the HIV-1 M group using env gene sequences, since traditional ML methods do not achieve optimal results due to a large imbalance in a sample size, where we find subtypes with less than 20 sequences and other subtypes with thousands of sequences. The solution is HIV-1 group subtype prediction based on env gene (HIV-1-M-SPBEnv), which uses artificial evolution techniques to generate synthetic training samples. The model was trained using 5,496 samples of the HIV Sequence DB from Los Alamos National Laboratory, saving one part to validate the synthetic data and another part as a set of validation of the final model. Before applying DL, sequence vectorization based on k-mer was used (with K = 6). The proposed architecture consists of an Autoencoder (AE) Convolutional 1D, which includes two ResNet in the encoder and two transposed ResNet in the decoder. The study applies L2 regularization (Ridge) in the training set, achieving 100% in all metrics for all subtypes in the validation set, facilitating viral monitoring and follow-up of these subtypes.

Another study is the one by Widowati [46], that employs a DNN to improve accuracy in HIV prediction and facilitate early identification in Indonesia. For this purpose, both laboratory indicators (CV, CD4,…) and demographic or socio-behavioral (age, residence, comorbidities,…), obtained from 17 hospitals in the country are used. The dataset includes complete information from 5,847 patients from 2020 to 2024. These were divided into training (70%), validation (15%) and test (15%), and hyperparameter optimization was achieved by using a Grid Search. The best model found obtained an accuracy of 94.2% and an F1-Score of 93.2%, surpassing ML methods such as RF or LR. SHAP analysis identified CD4+ cell count as the most important factor in predicting HIV, followed by the CD4/CD8 ratio, age and lymphocyte count. In addition, the model was used on a test set with missing and noisy data, showing consistent results exceeding, in both cases, 85% in accuracy.

We have seen that DL techniques are useful when wanting to work with longitudinal or genomic data that presents a significant complexity. Yet, we find relevant HIV information in textual form, for example, in clinical notes. Therefore, there is a need to use natural language techniques to deal with this information.

#### 4.3.4 NLP

In the context of HIV, NLP is applied on clinical notes or to analyse patient perceptions on social media.

In the area of comorbidities, Ridgway et al. [35] apply NLP on clinical notes to detect mental illness and substance use in people living with HIV. It uses data from 778 HIV-positive adults from the University of Medicine, Chicago (UCM), collecting 13,905 clinical notes. NLP algorithms were developed to identify words and phrases associated with mental illness (e.g., *Depression*, *Bipolar*, *Psychosis*) and substance use (e.g., *Cocaine*, *Heroin*, *Crack*, *Alcohol abuse*) in clinical notes. This was done in three steps. First, the sentences were tokenized and separated. The second step was to handle negation, using NegEx to identify and exclude occurrences of terms that were negated. Finally, a stemming algorithm was applied to recognize the different grammatical forms of a root word. Notes related to mental illness were identified with a Positive Predictive Value (PPV) of 98%, while for substance consumption the PPV was 92%, detecting documentation of mental illness in 54.0% (420/778) of patients and substance use in 18.1% (141/778). Of these, only 58.6% (246/420) had documentation of mental illness in at least one structured field of the EHR, while 73.8% (104/141) had documentation of substance consumption. This suggests that relying solely on the structured fields of the EHR may omit a significant number of patients.

In another study by Kakalou et al. [12], Twitter was analysed to identify perceptions and trends related to PrEP using data mining. After obtaining more than 32,000 tweets in English from September to December 2017, two data streams were created: one for patients using PrEP-related terms; and another for those using the drug trade names. Data analysis techniques included text mining, NLP (TF-IDF normalization) and topic modelling (using the Latent Dirichlet Allocation (LDA) algorithm). A sentiment analysis was also performed (using the AFINN and NRC lexicons) to determine the polarity of opinions expressed and to map emotions. Results indicated that it is possible to detect and obtain valuable information by monitoring PrEP discussions on Twitter, showing that the most common terms related to prevention or key populations at risk. General sentiment regarding PrEP was also positive, with confidence and anticipation being the most common emotions, while the main concerns related to stigma, cost of medication, and insurance coverage.

Finally, in the domain of diagnosis, we find the clinical trial of Chen et al. [22, 23], involving HIVST-chatbot, integrated in WhatsApp, is developed to provide real-time support to MSM to facilitate self-test for HIV. In this trial, 531 men are divided into two groups: first group (266) used the chatbot while the second group (265) interacted with human support. The percentages of men who took the test was very similar in both groups, exceeding 80%. It is important to highlight that the group using the chatbot had a significantly higher ratio of users who received advice (91.2% vs. 62.6%), probably due to 24 h availability of the chatbot, and the fact no prior appointment was required. In addition, this chatbot turned out to be more economical both in total cost as in the cost per assisted user. It proved to be an effective, scalable and cost-effective alternative to traditional services.

#### 4.3.5 LLM

The rise of LLMs has made it possible to develop tools capable of interacting with users by addressing a variety of questions in a dynamic way.

In the context of HIV we find the study by Tarhan et Ozdemir [55], which evaluates and compares the accuracy of ChatGPT-4o and Gemini when answering HIV-related questions obtained from public health sources, clinical guidelines and social networks. For this purpose, 156 HIV-related questions were posed to both models, obtained from the US Center for Disease Control and Prevention, several clinical guidelines and also questions derived from social networks. These questions were classified into four subgroups: general information, transmission, diagnosis and prevention and treatment. The responses were evaluated by two infectious disease specialists using a 4-point scale (1-“completely incorrect”, 4-“completely correct and sufficient”). The results obtained showed that ChatGPT had a completely correct response rate of 81.4% compared to 71.8% for Gemini. In relation to question categories, they achieved more than 80% in almost all except for “Prevention and Treatment” where neither model reached 70% accuracy. Reproducibility of both models was also found to be high, exceeding 90%.

Another study by Zhou et al. (2025, preprint) [54], proposes a pipeline that uses an LLM to analyse clinical notes and determine a patient’s eligibility for additional HIV testing. Building on Google’s MedGemma model, pretrained extensively in medical texts, and using data from the Erasmus HIV Dataset assigns test inclusion (1) or exclusion (0) labels. The pipeline embeds the EuroTEST guide directly into the MedGemma model *prompt* to make decisions consistent with current HIV screening best practices. As an experimental setup two prompts are proposed: a Simple Prompt (SP) with a 3-step Chain-of-Thought (CoT) reasoning, and a 9-step Complex Prompt (CP), with more detailed guidelines. In addition, different strategies were evaluated for the aggregation of results such as choosing the first prediction or the output with more tokens. Results showed that the pipeline achieved a high overall accuracy while maintaining a low false negative rate (high sensitivity). In addition, MedGemma-SP outperformed MedGemma-CP in terms of sensitivity, with autoconsistency emerging as the best aggregation strategy.

#### 4.3.6 Evaluation of approaches

Reviewed studies show clear correspondence between data types, clinical objective and technique used. Statistical and supervised learning methods predominate in structured scenarios with known labels, while unsupervised learning is used to find population subgroups with common characteristics. In turn, Deep Learning, NLP and LLMs are employed when working with unstructured data and high dimensionality, since the power of neural networks permit addressing these singularities.

Although predictive performance was frequently reported across the reviewed ML and DL studies, important methodological differences were observed regarding validation strategies, external validation, dataset size, synthetic data usage, and model interpretability. To provide context for the reported performance metrics, Table 3 summarises these methodological characteristics for the predictive AI studies included in this review.

**Table 3:** Methodological assessment of predictive ML, DL and NLP based studies.

Methodological indicator		Studies
External validation		[11, 62]
Dataset size ($N^{o}$ of patients)	<1,000	[17, 26, 30, 32, 35, 39, 43, 45, 53]
	1,000–5,000	[19, 24, 25, 29, 33, 34, 36, 37, 47, 59, 63]
	>5,000	[11, 13, 31, 38, 44, 46, 49, 52, 60–62]
Synthetic data used		[40, 44]
Evaluation	Cross validation	[18, 24–26, 29, 33, 34, 44, 47, 49, 61, 63]
	Hold-out	[11, 17, 40, 43, 45, 46, 62]
	Explainability	[34, 43, 46]

For NLP based studies that evaluated clinical notes, dataset size taken was based on the number of patients included in clinical notes.

To complement the literature synthesis, we performed a methodological characterisation of the predictive machine learning and deep learning studies included in this review. Rather than conducting a formal risk-of-bias assessment, we summarised key aspects related to model robustness and clinical applicability, drawing inspiration from the reporting and evaluation domains proposed in TRIPOD-AI and PROBAST. Specifically, we examined external validation, dataset size, the use of synthetic data, validation strategies, and model interpretability because they are directly related to the generalisability, reliability, and clinical applicability of predictive models.

The methodological assessment revealed important limitations in the robustness of predictive AI studies in HIV research. While dataset sizes were generally heterogeneous and reasonably distributed across scales, only a small number of studies performed external validation, while most relied on internal validation procedures as cross-validation, which may lead to optimistic performance estimates in the absence of external testing. Similarly, explainability techniques were inconsistently adopted, and only a few studies reported the use of synthetic data to address data scarcity or class imbalance. These findings suggest that the predictive performance reported in the literature should be interpreted with caution, as methodological robustness does not always match the reported accuracy metrics.

These observations reinforce the need of a task-oriented framework that links HIV-related clinical problems with appropriate artificial intelligence methodologies, aiming to support more robust and clinically meaningful model development. Also, the diversity of AI approaches and varied HIV areas of interest suggest the need to establish a clear reference framework to support researchers and clinicians in advancing this medical area.

### 4.4 A task-oriented framework for applying AI in HIV

This section proposes a task-oriented framework that aims to help practitioners and researchers select the most appropriate AI approach according to the clinical task faced, considering in each case the particular type of data and its technical complexity. This framework is based on the analysis of the reviewed studies, assessing the techniques used in each of them, their performance, effectiveness and possible limitations.

In Table 4, we can find an overview on which approaches stand out at each clinical domain for each specific task. A set of recommendations for each phase of clinical management are described below.

**Table 4:** Task-oriented framework for selecting AI techniques based on clinical tasks in the different clinical domains of HIV.

HIV medical focus	Clinical task	AI technique					
		Statistical approaches	Supervised	Non supervised	DL	NLP	LLM
Prevention	∙ Identification of PrEP candidates	–	✓	–	–	✓	–
	∙ Infection risk analysis	–	✓	–	✓	✓	–
	∙ Screening/characterization of HIV	–	✓	✓	✓	–	✓
	∙ Information for HIV prevention	–	–	–	✓	✓	✓
Diagnosis	∙ HIV positive prediction	–	✓	–	✓	–	–
	∙ Risk identification using clinical notes	–	✓	–	–	✓	✓
Treatment	∙ Response to ART	✓	✓	–	–	–	–
	∙ Resistance to antibodies and inhibitors	✓	✓	–	✓	–	–
	∙ Treatment adherence and retention	–	✓	–	–	✓	✓
Progression	∙ LLV/VF progression prediction	✓	✓	–	–	–	–
	∙ Subgroup identification based on patterns	–	✓	✓	–	–	–
Comorbidities Outcomes	∙ Risk of chronic diseases appearance	✓	✓	–	–	–	–
	∙ HIV Comorbidities prediction	–	✓	–	✓	–	–
	∙ Detection of mental illness and socio-behavioural pathologies	–	–	✓	–	✓	–
	∙ Analysis of HIV genes	–	–	–	✓	–	–

#### 4.4.1 Prevention

This clinical domain includes tasks such as the identification of PrEP candidates, infection risk analysis, population screening and the gathering of information in relation to prevention. For the identification of PrEP candidates, supervised models on structured clinical data are recommended [11, 52, 61, 62], as well as the development of chatbots to generate PrEP demand [14], and the use of NLP to examine perceptions and trends in social networks [12]. In the HIV risk analysis, non-linear models based on DL such as Graph Convolutional Network (GCN), based on graphs, are suitable for working with textual information obtained from social networks [17]. When it comes to characterization and screening of the disease, unsupervised techniques are useful [13], especially those based on hierarchical clustering with euclidean distance to capture homogeneity in groups of patients. Neural network-based approaches (e.g., DNN) can improve prediction accuracy [46], and LLMs such as MedGemma on clinical notes [54] are also recommended to determine whether a patient may require additional testing. Finally, the use of NLP techniques allow working with text and help provide relevant information about HIV, using models such as LSTM or BiGRU to develop chatbot systems [15, 16].

#### 4.4.2 Diagnosis

Here we find two main tasks: predicting HIV-positives from structured data and identifying risks in patient clinical notes. For HIV positive prediction the use of supervised learning techniques such as SVM or RF is recommended, as well as deep models capable of modelling complex relationships over numerical variables [18, 21]. In contrast, with unstructured information as text in the task of risk identification, NLP techniques such as LDA are used to identify topics or TF-IDF to find key terms about clinical notes [39]. Pre-trained LLMs like ${\mathrm{RoBERTa}}_{\mathrm{Bio}}$, are also robust compared to classic ML algorithms [19].

#### 4.4.3 Treatment

AI impact on HIV treatment is focused on evaluation of response to ART, antibodies and virus inhibitors resistance, and the analysis of treatment adherence and retention. In the evaluation of response of patients under ART, classical statistical models are still widely recommended such as Lasso regression along with Cox [59]; it is also possible to use supervised models such as XGBoost, RF or GB to capture non-linear relationships [24]. Conversely, when we deal with genomic data, such as when evaluating HIV inhibitors or antibodies resistance, the most suitable techniques are based on DL as CNN or BRNN or variations of statistical approaches such as logistic regression [41]. Finally, when addressing treatment adherence and retention it is interesting to use supervised models such as GB along with balancing techniques such as SMOTE to treat imbalance. In addition, we can apply NLP techniques on clinical notes using negation tokens to distinguish the presence or absence of pathologies or use n-grams to better capture the terms [53]. Also, the use of LLMs such as Google Bard (now Gemini) allows for evaluation of answers to treatment-related questions [55, 56].

#### 4.4.4 Progression

This clinical domain is important due to the appearance of pathologies such as LLV or VF. In this sense, AI techniques are used to predict the progression of the disease towards these adverse events, as well as to identify subgroups of patients based on longitudinal progression patterns. In LLV or VF prediction, classical statistics models such as Cox’s proportional risk or multivariate logistic regression are very useful for these risk analysis [30, 31]. Supervised techniques such as RF or XGBoost are also suitable, for example when analysing progression from HIV to AIDS [29, 44]. Another task is the identification of patient subgroups. Here, supervised techniques as RF or SVM support as a pre-clustering step, obtaining a compressed representation of the data [63]. Next, hierarchical clustering algorithms are employed using distances such as the euclidean or dynamic time warping (DTW) to be able to determine various clusters indicating the different rates of disease progression [32].

#### 4.4.5 Cormorbidities and outcomes

Finally, at this domain we find tasks such as the identification of the risk of acquiring chronic diseases, prediction of HIV co-morbidities, detection of mental illnesses and socio-behavioural pathologies or the HIV genes analysis. When assessing the risk of developing chronic diseases like Diabetes Mellitus or hyperlipidemia it is recommended to use both classical (e.g., Cox) [60] and ML models like RF and LightGBM, which, used along with explainable methods like SHAP, make it possible to identify the most important variables [34]. In the prediction of HIV comorbidities the use of ML algorithms such as XGB, and DL-based techniques such as MLP or TabNet for multi-tag prediction are recommended [33]. In the detection of mental illnesses and socio-behavioural pathologies, we can employ agglomerative hierarchical clustering [47] if we have numerical data or NLP techniques if what we have is text, as in the case of clinical notes [35]. Among these latter techniques are NegEx to handle negation and tools like Stanford CoreNLP to tokenize and separate sentences. Finally, the last task is HIV gene analysis, where DL-based techniques are desirable, mainly based on mechanisms of attention or Autoencoders. Here, we find implementations such as DeepHINT or HIV-1-M-SPBEnv, which stand out for their ability to work with this type of longitudinal data [40].

In order to simplify as much as possible the selection of techniques, we recommend in Table 5 some algorithms and specific tools for each AI approach, based not only on what literature signals, but due to real suitability for each clinical scenario, data types and effectiveness in similar tasks in other fields.

**Table 5:** Recommended techniques in the context of HIV.

AI approach	AI techniques
Statistical approaches	Cox proportional risk model, Multivariant logistic regression
Supervised ML	RF, XGBoost, LightGBM
Unsupervised ML	Agglomerative Clustering + euclidean or DTW distance
DL	MLP, TabNet, LSTM, DNN
NLP	TF-IDF, LDA, NegEx
LLM	MedGemma, ${\mathrm{RoBERTa}}_{\mathrm{Bio}}$

## 5 Discussion

This study proposes a task-oriented framework to facilitate the use of Artificial Intelligence in the context of HIV, assisting researchers in this field (both clinicians and engineers). After analysing the existing literature in the different clinical application domains, data types and sources, and AI techniques, we provide a tool that links common clinical HIV-related tasks with appropriate AI approaches, in order to select a suitable AI approach based on the clinical context and available data.

### 5.1 Comparison with prior literature

Compared with previous reviews in the field, this work extends the existing literature by moving from descriptive evidence synthesis to a decision-oriented methodological framework. Previous reviews have organised AI applications according to different perspectives. For example, Xiang et al. [51] structured the literature around four HIV prevention-intervention objectives (prevention, testing, treatment, and response), whereas Ngcobo et al. [20] synthesised studies according to four stages of HIV care and additionally described the machine learning methods most frequently employed within each domain. Similarly, Ge et al. [58] analysed AI-based HIV risk prediction by decomposing the modelling workflow into sequential stages, from data acquisition to model validation.

While these reviews provide valuable overviews of AI applications from clinical or methodological perspectives, they primarily describe how AI has been used in previous studies. In contrast, the framework proposed in this work integrates clinical application domains, data characteristics, and AI methodologies to support the selection of appropriate techniques for specific HIV-related tasks. Rather than classifying existing applications, it provides a task-oriented structure intended to guide future methodological decisions in both research and clinical informatics.

### 5.2 Key findings and methodological gaps

The analysis of the literature also reveals some gaps in the application of AI across the different HIV clinical domains. On the one hand, by attending to these domains, we observe a large proportion of studies related to prevention and some comorbidities, while other domains like diagnosis or progression have less representation in the literature despite their potential importance in the management of HIV. On the other hand, from a methodological perspective, most studies focus on supervised machine learning using structured data or the use of NLP or chatbots with unstructured data, while other AI approaches such as clustering, semi-supervised learning, or even reinforcement learning, are still open to more investigation.

### 5.3 Clinical and translational implications

Beyond its application in research, this task-oriented framework can be useful in clinical practice. Proper selection of AI algorithms and techniques can contribute, for example, to early detection of poor treatment adherence, virological failure or appearance of non-definitory AIDS comorbidities. With this framework, it is possible to prioritize resources and provide personalized treatments through a conceptual framework that allows different techniques to be implemented at the medical care level, translating methodological advances into practical application.

Translation into clinical practice requires careful consideration of model interpretability, explainability, robustness, and integration into existing clinical workflows. The adoption of AI systems in HIV care requires regulatory consideration and future compliance. The trust of clinical experts especially in high risk decision making contexts is crucial.

### 5.4 Limitations

There are some limitations to this study. Firstly, although the literature search was structured and reproducible, it does not constitute a fully systematic review, and therefore some relevant studies may not have been captured.

Secondly, when applying the proposed framework mapping AI techniques to clinical tasks, it is possible that data used may be imbalanced or some bias is introduced in certain classifications, such that empirical validation of techniques would be necessary. Nevertheless, this work is presented as a methodological starting point.

Thirdly, only studies published in English were included, which may have introduced language bias and reduced the representation of research conducted in non-English-speaking settings. This is particularly relevant in the context of HIV, where a substantial proportion of the global disease burden is concentrated in low- and middle-income countries. Although many studies from non-English-speaking countries are disseminated through English language journals, excluding publications in other languages may have led to the omission of locally developed AI solutions, healthcare workflows, or data resources that are not widely represented in the international literature.

Finally, the proposed framework has to be continuously redefined and updated, adapting it accordingly to the challenges presented and new evolving models.

### 5.5 Future directions

Future research should focus on validating the proposed framework in applied clinical and research settings. In particular, empirical evaluation of whether this decision-oriented structure improves methodological selection or predictive performance in HIV-related applications would be valuable.

Also, there is a need for a global HIV cohort, the further development of explainable methods, and the management of bias in data.
